# An annotated checklist of the Cook Islands psyllids with keys to the species and two new records (Hemiptera, Psylloidea)

**DOI:** 10.3897/zookeys.811.28829

**Published:** 2018-12-31

**Authors:** Francesco Martoni, Samuel D. J. Brown

**Affiliations:** 1 Bio-Protection Research Centre, Lincoln University, Lincoln 7647, New Zealand Lincoln University Lincoln New Zealand; 2 Agriculture Victoria Research, AgriBio Centre, 5 Ring road, Bundoora 3083, Victoria, Australia Agriculture Victoria Research Victoria Australia; 3 The New Zealand Institute for Plant and Food Research Limited, Private Bag 92169, Auckland Mail Centre 1142, New Zealand The New Zealand Institute for Plant and Food Research Limited Auckland New Zealand

**Keywords:** Jumping plant lice, Pacific Islands, Polynesia, Rarotonga, Sternorrhyncha

## Abstract

An annotated checklist of the psyllids of the Cook Islands is presented. The presence of *Syntomozatahuata* (Klyver, 1932) and *Triozaalifumosa* Klyver, 1932 in the archipelago, based on new material collected, is reported for the first time. This is the first record from these islands of the genus *Syntomoza* and the family Liviidae. An identification key to the psyllid species known from the Cook Islands is provided, and their origin and provenance are discussed in relation to their biogeographic implications.

## Introduction

The superfamily Psylloidea (Hemiptera: Sternorrhyncha) is composed of almost 4000 described species worldwide (Ouvrard 2018). These include taxa used for biological control, such as *Arytainillaspartiophila* (Förster, 1848) released in New Zealand to control Scotch broom, *Cytisusscoparius* (L.) Link (Fabaceae) (Syrett et al. 2007), and also a number of species listed as pests (EPPO/CABI 1997). Among these, a few taxa are known to vector plant pathogenic bacteria (e.g. Munyaneza 2007, 2014). Such a broad range of ecological functions ensures that psyllids’ movement between countries is of interest. For example, a recent study implemented modelling analyses to assess the risk and predict the spread of the pest species *Russellianasolanicola* Tuthill, 1959, the South American potato psyllid, to several countries where it is not yet present (Syfert et al. 2017). Similarly, the recent establishment of the tomato/potato psyllid *Bactericeracockerelli* (Šulc), vector of *Candidatus* Liberibacter solanacearum and agent of the zebra chips disease, has caused great economic losses in New Zealand (Vereijssen et al. 2018). Therefore, understanding psyllid distributions is fundamental to assess the risk associated with new invasions. In recent years, research on psyllid biodiversity has been conducted in a number of regions and islands of the Austro-Pacific. These include the description of taxa in Australia (Taylor et al. 2016, Taylor 2018), the reclassification of *Pariaconus* Enderlein, 1926 and *Swezeyana* Caldwell, 1940 in the Hawaiian Islands (Percy 2017, 2018) and reports of the arrival of alien species in Australia (Taylor and Kent 2013), New Zealand (Thorpe 2013, Martoni et al. 2016, Martoni et al. 2018) and French Polynesia (Claridge et al. 2014). However, the psyllid fauna of most other Pacific Islands has not been updated for many years (Ouvrard 2018).

The first report on the psyllid fauna of the Cook Islands appears in Hodkinson’s checklist of the Austro-Oriental and Pacific area that listed three species: *Mesohomotomahibisci* (Froggatt, 1901); *Leptynopterasulfurea* Crawford, 1919; and *Triozavitiensis* Kirkaldy, 1907 (Hodkinson 1983). An additional species, *Heteropsyllacubana* Crawford, 1914, was reported a few years later (Muddiman et al. 1992). The most recent addition was a *Trioza* species similar to *T.zimmermani* Tuthill, 1942, identified by P. Dale and recorded in the online Cook Island Biodiversity and Natural Heritage database (McCormack 2007).

The geographical location of the Cook Islands puts them in a central position between French Polynesia and other countries such as Samoa, Tonga, Fiji, and New Zealand. This makes this small archipelago important for evaluating biogeographic hypotheses and testing theories of biological dispersal within the Pacific. Additionally, due to the high movement of people and produce between the Cook Islands, New Zealand and Australia, understanding the biodiversity of the Cook Islands allows evaluation of potential biosecurity risks for New Zealand or Australian agriculture.

For these reasons, recent field collections from the Cook Islands presented in this work have contributed to updating our knowledge of the psyllid biodiversity of the Islands, with the discovery of two additional taxa: *Syntomozatahuata* (Klyver, 1932), and *Triozaalifumosa* Klyver, 1932, both originally described from French Polynesia (Marquesas) (Klyver 1932).

## Materials and methods

Specimens were collected by SDJB on the island of Rarotonga, Cook Islands, in March and April 2017. Collections were made by beating host foliage over a beating tray. Insects were stored in propylene glycol until morphological identification was performed. Photographs were taken using a Nikon DS-Ri2 camera connected to a Nikon SMZ25 microscope. Pictures presented in the plates are the result of stacking photographs using the software Nikon NIS-Elements D v4.5 resulting in a single image with an extended depth of field. Plates were prepared using GIMP version 2.8.14. For closer examination, two specimens of each species (male and female) were mounted on microscope slides following the protocol of Taylor et al. (2016). Morphological terms follow Taylor et al. (2011) and wing venation follows Hodkinson and White (1979) and Hollis (1984). Psyllid specimens from the recent field collection have been deposited in the New Zealand Arthropod Collection (**NZAC**, Manaaki Whenua Landcare Research, Tamaki, Auckland), and the Lincoln University Entomology Collection (**LUNZ**, Canterbury). Plants were identified by SDJB using Sykes (2016). Specimens of the host plants collected at the same time as insect specimens were deposited in the Allan Herbarium (Landcare Research, Lincoln, New Zealand), with catalogue numbers CHR644589 (*Homaliumacuminatum*), CHR644590 (*Weinmanniasamoensis*), and CHR644584 and CHR644585 (*Metrosideroscollina*). Paratype specimens of *T.alifumosa* and *T.alipellucida* Klyver, 1932 were examined in the Bernice Pauahi Bishop Museum (**BPBM**, Honolulu, Hawaii).

## Identification of the newly reported species

### Syntomoza tahuata

Taxon classificationAnimaliaHemipteraLiviidae

(Klyver, 1932)

Figures 1–10 , 23

#### Material examined.

4 females, 10 males. This species was collected on two separate occasions on Rarotonga: on 15 April 2017 on Te Manga at elevations between 540 m and 560 m, collected from two host plants: from *Weinmanniasamoensis* A.Gray (Cunionaceae) (five specimens) and from *Freycinetiawilderi* Martelli ex Wilder (Pandanaceae, plant specimens not collected) (two specimens), and on 17 April 2017 in the Avana Valley around 70 m elevation, from the foliage of a fallen *Homaliumacuminatum* Cheeseman (Salicaceae) (seven specimens collected, with several more observed). Three additional specimens collected around Avatiu in November 1979 by NLH Krauss were located in the Bishop Museum.

**Figures 1–10.**
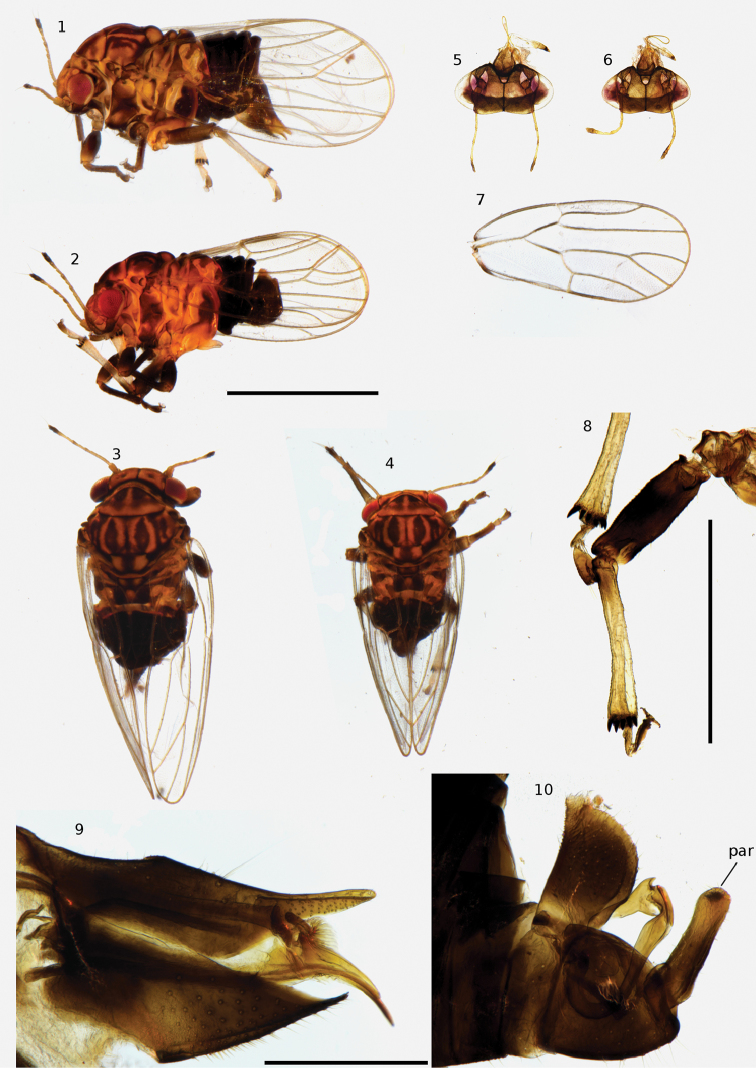
*Syntomozatahuata*. **1** lateral habitus of female **2** lateral habitus of male **3** dorsal habitus of female **4** dorsal habitus of male **5** head of female, dorsal view **6** head of male, dorsal view **7** wing of male **8** mesotibia of male **9** terminalia of female, lateral view of left side **10** terminalia of male, lateral view of left side. Abbreviation: par = paramere. Scale bars: 1 mm (**1–7**); 0.5 mm (**8**); 0.25 mm (**9, 10**).

#### Measurements.

Measurements are in mm (n = 3 ♂, 2 ♀ unless reported differently in brackets). Length of body (vertex to terminalia) ♂ 0.98–1.30 (n = 2), ♀ 1.17–1.53; length of body (vertex to apex of folded wings) ♂ 1.72–1.88 (n = 2), ♀ 2.21–2.22; width of head (HW) ♂ 0.53–0.60 (n = 2), ♀ 0.63–0.65; length of genal processes (GCL) ♂ 0.10 (n=1), ♀ 0.12; length of vertex (VL) ♂ 0.12–0.18 (n = 2), ♀ 0.18–0.19; width of vertex (VW) ♂ 0.30–0.35 (n = 2), ♀ 0.34–0.38; length of antenna (AL) ♂ 0.40–0.49 (n = 2), ♀ 0.44–0.57; length of fore wing ♂ 1.40–1.49, ♀ 1.71–1.77; width of fore wing ♂ 0.63–0.68, ♀ 0.75–0.85; length of vein Rs ♂ 0.82–0.87, ♀ 1.02–1.04; length of vein M (M) ♂ 0.44–0.46, ♀ 0.52–0.53; length of vein M1+2 (M1) ♂ 0.36–0.40, ♀ 0.48–0.51; marginal width of cell m1 ♂ 0.18–0.20, ♀ 0.26–0.27; marginal width of cell cu1 ♂ 0.50–0.54, ♀ 0.62–0.63; length of vein Cu1b ♂ 0.11–0.14, ♀ 0.13–0.16; length (height) of proctiger (PL) ♂ 0.21–0.24 (n = 2); length of paramere ♂ 0.17–0.19 (n = 2); length of proximal aedeagal segment ♂ 0.19 (n = 1); length of distal aedeagal segment ♂ 0.09 (n = 1); length of proctiger (PL) ♀ 0.44–0.52; length of circum-anal ring (CL) ♀ 0.16–0.20; length of subgenital plate (SL) ♀ 0.35–0.46.

#### Description.

The stout body shape, and the distinct dorsal patterning of orange stripes on a black background makes this psyllid readily recognised within the Cook Island fauna. This psyllid was identified using the original description (Klyver 1932) and the subsequent reclassification that attributed this species to the genus *Syntomoza* Enderlein, 1921 (Burckhardt and Mifsud 2003). Other features that allow it to be placed in *S.tahuata* include the greatly modified tergites and the secondary groups of small teeth at the apex of the posterior tibiae in both sexes (Figure 8), which are characteristic of this genus, together with the strongly inclined head (at about 90° to the longitudinal body axis; Figures 1, 2). Furthermore, the female terminalia which are pronouncedly down-turned at about 45° (Figure 1), the shape of the male parameres (Figure 10), and wing shape and venation (Figure 7) allowed identification of this species as per the description and figures presented by Klyver (1932).

### Trioza alifumosa

Taxon classificationAnimaliaHemipteraLiviidae

Klyver, 1932

Figures 11–20 , 26

#### Material examined.

11 females, 8 males. A single population of this species was collected on Rarotonga, on the summit of Raemaru at an elevation of 380 m. On 16 March 2017 all 19 individuals were collected from a single plant of *Metrosideroscollina* (J.R.Forst. and G.Forst.) A.Gray.

#### Measurements.

Measurements are in mm (n = 2 ♂, 3 ♀ unless reported differently in brackets). Length of body (vertex to terminalia) ♂ 1.30–1.45, ♀ 1.60–1.78; length of body (vertex to apex of folded wings) ♂ 2.57–2.81, ♀ 2.86–3.10; width of head (HW) ♂ 0.50–0.53, ♀ 0.52–0.57 (n = 2); length of genal processes (GCL) ♂ 0.09–0.14 ♀ 0.10–0.13 (n = 2); length of vertex (VL) ♂ 0.21, ♀ 0.20–0.25 (n = 2); width of vertex (VW) ♂ 0.31–0.32, ♀ 0.32–0.33 (n = 2); length of antenna (AL) ♂ 0.78–0.79, ♀ 0.81–0.85 (n = 2); length of fore wing ♂ 2.27–2.28, ♀ 2.38–2.57 (n = 2); width of fore wing ♂ 0.83–0.86, ♀ 0.85–0.96 (n = 2); length of vein Rs ♂ 0.91–0.99, ♀ 1.00–1.08 (n = 2); length of vein M (M) ♂ 1.11–1.12, ♀ 1.15–1.24 (n = 2); length of vein M1+2 (M1) ♂ 0.44–0.48, ♀ 0.54–0.56 (n = 2); marginal width of cell m1 ♂ 0.28–0.32, ♀ 0.38 (n = 2); marginal width of cell cu1 ♂ 0.40–0.42, ♀ 0.42–0.44 (n = 2); length of vein Cu1b ♂ 0.23–0.25, ♀ 0.21–0.25 (n = 2); length (height) of proctiger (PL) ♂ 0.15–0.20; length of paramere ♂ 0.11–0.13; length of proximal aedeagal segment ♂ 0.17 (n = 1); length of distal aedeagal segment ♂ 0.16 (n = 1); length of proctiger (PL) ♀ 0.30–0.51; length of circum-anal ring (CL) ♀ 0.10–0.13; length of subgenital plate (SL) ♀ 0.29–0.34.

**Figures 11–20.**
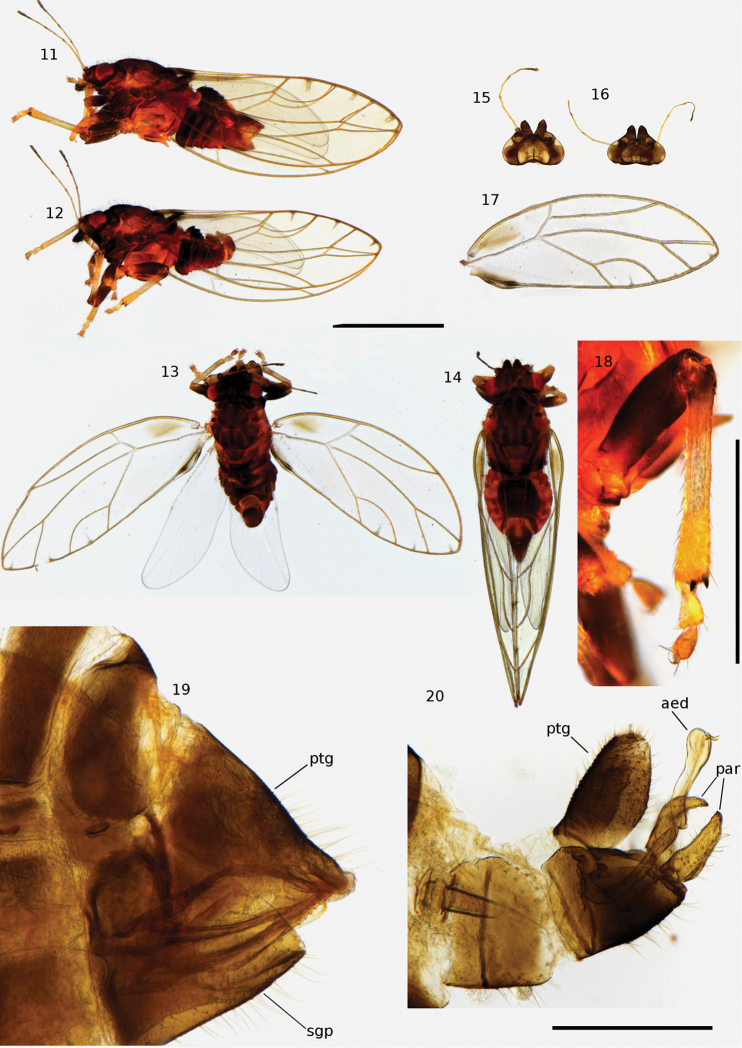
*Triozaalifumosa*. **11** lateral habitus of female **12** lateral habitus of male **13** dorsal habitus of male **14** dorsal habitus of female **15** head of female, dorsal view **16** head of male, dorsal view **17** wing of male **18** mesotibia of male **19** terminalia of female, lateral view of left side **20** terminalia of male, lateral view of left side. Abbreviations: aed = aedeagus, par = paramere, ptg = proctiger, sgp = subgenital plate. Scale bars: 1 mm (**11–17**); 0.5 mm (**18**); 0.25 mm (**19, 20**).

#### Description.

This psyllid can be identified by the following combination of characters: habitus as in Figures 11–14, with a dark brown colour, fore wings with an infuscate spot in the apical costal cell as in Figures 13, 17, female proctiger short and bearing setae on the subgenital plate (Figure 19); male parameres elongate, slightly back-turned apically and bearing setae (Figure 20). Both this species and *T.alipellucida* Klyver, 1932, were described from material collected on *Metrosideroscollina*. The evenly dark colouration of the dorsal surface and head, the presence of an infuscate spot in the apical costal cell (c+sc), the rounded but elongated shape of the aedeagus, the elongated shape of the male proctiger and the slightly back-turned parameres lead us to place it in *T.alifumosa*. *Triozaalipellucida* differs from *T.alifumosa* by most specimens having a wide lighter brown stripe on the pronotum, not having an infuscate spot at the base of the forewing, and for a shorter male proctiger associated with parameres that are not as back-turned. The morphological distinction between *T.alifumosa* and *T.zimmermani* appears more immediate, with the latter presenting light stripes dorsally on a dark brown abdomen and having hyaline wings without any dark spot in the cell c+sc (Tuthill 1942).

## Checklist of the Cook Islands psyllids

The following checklist includes all species known to be present in the Cook Islands. Information on their taxonomy is reported together with their worldwide distribution and host plant associations. For species of socio-economic interest, such as pests, basic information on their biology is summarised.

### Family Carsidaridae

#### Mesohomotoma hibisci

Taxon classificationAnimaliaHemipteraCarsidaridae

(Froggatt, 1901)

Figures 21 , 28

Tyora hibisci Froggatt, 1901: 287.
Udamostigma hibisci (Froggatt); Enderlein 1910: 138.
Mesohomotoma hibisci (Froggatt); Crawford 1925: 32.

##### Distribution.

Reported on the Cook Islands by Hodkinson (1983). Known from Rarotonga and Mangaia. Other locations include: Australia (Hollis 2004), Africa [Cameroon, Democratic Republic of the Congo, Kenya, Madagascar, Seychelles, South Africa, Tanzania, Uganda and Zimbabwe (Yana et al. 2015; Burckhardt and Van Harten 2006)], Asia [Chagos archipelago, China, India, Japan, Malaya, Malaysia, Mauritius, Philippines, Ryukyu Islands, Singapore, Yemen (Hodkinson 1983, Hodkinson 1986, Burckhardt and Van Harten 2006, Percy 2017)], Pacific Islands [Bismarck Archipelago, Caroline Islands, Fiji, French Polynesia (Australs, Societies, Marquesas), Gilbert Islands, New Caledonia, Palau, Tonga, Solomon Islands, Vanuatu (Hodkinson 1983)].

##### Host plant.

*Hibiscus* species, especially *H.tiliaceus* L. (Malvaceae).

##### Common name.

Hibiscus (woolly) psyllid (David Hockings 2013).

##### Remarks.

**Figure 21.**
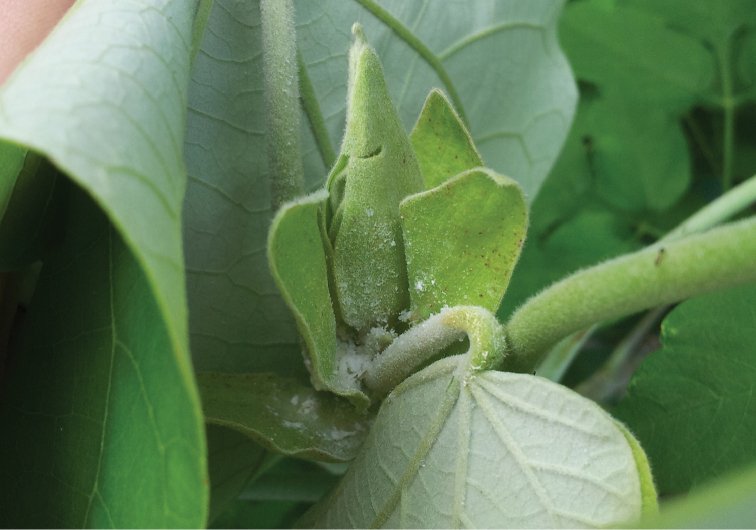
*Mesohomotomahibisci* nymphs and adult on *Hibiscustiliaceus* on Rarotonga, showing white waxy exudates formed by the nymphs.

the genus *Mesohomotoma* Kuwayama was reviewed by Hollis (1987). The species included in the genus have a lot of variation between populations, and subtle differences between species. Although Hollis (1987) suspected all nominal taxa may represent a single species, he did not formally synonymise them, recommending that further research into their biology and hostplants be undertaken to further investigate species boundaries in the genus. This species breeds in the tips of *Hibiscustiliaceus* branches. The nymphs produce filamentous exudates, which forms a woolly coating on the leaves and stem of the plant (Figure 21). *Mesohomotomahibisci* is considered a pest (David Hockings 2013).

### Family Liviidae

#### Syntomoza tahuata

Taxon classificationAnimaliaHemipteraLiviidae

(Klyver, 1932)

Figures 1–10 , 23

Anomoterga tahuata Klyver, 1932: 94.
Syntomoza tahuata (Klyver); Burckhardt and Mifsud 2003: 17.

##### Distribution.

Reported on the Cook Islands in the present study. Known only from Rarotonga. Other locations include: French Polynesia (Marquesas) (Klyver 1932).

##### Host plant.

No host plants have been previously proposed (Burckhardt and Mifsud 2003; Ouvrard 2018). In June 2002, however, Percy (pers. comm.) collected a high number of adult specimens (> 30) from *Weinmanniaparviflora* in French Polynesia (Marquesas) with no specimens found on surrounding plants.

### Family Psyllidae

#### Heteropsylla cubana

Taxon classificationAnimaliaHemipteraPsyllidae

Crawford, 1914

Figure 25

Heteropsylla cubana Crawford, 1914.

##### Distribution.

Reported on the Cook Islands by Hodkinson (1983). Known only from Rarotonga. Other locations include: Australia (Muddiman et al. 1992), America [Bahamas, Bermuda, Brazil , Central America, Chile, Colombia, Costa Rica, Cuba, Dominican Republic, Ecuador, El Salvador, Guatemala, Jamaica, Mexico, Nicaragua, Panama, Peru, Suriname, Trinidad and Tobago, USA (Brown and Hodkinson 1988, Burckhardt and Queiroz 2012, Hodkinson and White 1981, Hodkinson 1988, Hodkinson and Muddiman 1993, Muddiman et al. 1992, Olivares and Burckhardt 2002, Percy et al. 2012)], Africa [Burundi, Cameroon, Kenya, KwaZulu-Natal, Mauritius, Mpumalanga, Reunion, Tanzania, Uganda, Zimbabwe (FAO 1994, Dzokou et al. 2009, Matimati et al. 2009, Muddiman et al. 1992, Olckers 2011)], Asia [Bangladesh, Cambodia, China, Christmas Islands, India, Indonesia, Japan, Malaysia, Mariana Islands, Nepal, Ryukyu Islands, Philippines, Sri Lanka, Taiwan, Thailand, Vietnam (Muddiman et al. 1992, Martin and Lau 2011, Inoue and Miyatake 2001, Geiger and Gutierrez 2000)], Pacific Islands [Fiji, French Polynesia (Australs), Guam, Haiti, Hawaiian Islands, New Caledonia, Niue, Papua New Guinea, Samoa, Solomon Islands, Tonga (Beardsley and Uchida 1990, Claridge et al. 2014, Muddiman et al. 1992, FAO 1994)], Europe [Ireland (Muddiman et al. 1992)].

##### Host plant.

*Leucaenaleucocephala* (Lam.) de Wit (Fabaceae).

##### Common name.

Leucaena psyllid (Asadi et al. 2011).

##### Remarks.

*Heterpsyllacubana* is considered an agricultural pest both in the Asia-Pacific area and in Africa (FAO 1994). The biological control agent that has been used most and with better results is the parasitoid *Psyllaephagusyaseeni* Noyes, 1990 (Encyrtidae), but *Curinuscoeruleus* Mulsant, 1850 (Coccinellidae) and *Tamarixialeucaenae* Boucek, 1988 (Eulophidae) have been used as well (Geiger and Gutierrez 2000).

##### Biology.

The biology and life cycle of *H.cubana* is reported here with the intent of summarising information (mostly from Showler and Melcher 1995 and CABI 1990) that may be relevant for a better understanding of this pest species. The incubation period for eggs is generally 2–5 days. Immature stages grow from the egg through five instars to adulthood in 10–20 days. Nymphs feed at first gregariously near the oviposition site and then, more and more solitarily, they colonise and feed on other parts of stems, branches, and petioles of young leaves. Generations are overlapping, and longevity of adults is on average 14.5 days for females and 9.7 days for males. Mating can occur more than once for both males and females (Rauf et al. 1990) and eggs are laid in groups on very young shoots, often covering the whole leaflet. Each female can produce 300–500 eggs throughout a lifetime and can lay as many as 60 eggs in one day. *Heteropsyllacubana* is diurnal, and flight of adults can occur in the morning and afternoon.

### Family Triozidae

#### Leptynoptera sulfurea

Taxon classificationAnimaliaHemipteraTriozidae

Crawford, 1919

Figure 22

Leptynoptera sulfurea Crawford, 1919: 147.

##### Distribution.

Reported on the Cook Islands by Hodkinson (1983). Known only from Rarotonga. Other locations include: Australia (Hollis 2004), Asia [China, Chagos Islands, Cocos Islands, India, Indonesia, Japan, Malaysia, Philippines, Ryukyu Islands, Singapore, Sulawesi, Taiwan, Thailand (Martin and Hollis 1992, Hodkinson 1983, 1986, Neville et al. 2015)], Pacific Islands [Caroline Islands, Fiji, French Polynesia (Australs), Guam, Hawaiian Islands, Mariana Islands, New Caledonia, Palau, Papua New Guinea, Tonga (Hodkinson 1983, Martin and Hollis 1992, Percy 2017].

##### Host plant.

*Calophylluminophyllum* L. (Calophyllaceae).

##### Remarks.

*Leptynopterasulfurea* forms galls along the leaf margins of *Calophylluminophyllum* (Neville et al. 2015), a tree of particular significance for Cook Islanders in that the trunks were preferentially used for building canoes (Hiroa 1927).

#### Trioza alifumosa

Taxon classificationAnimaliaHemipteraTriozidae

Klyver, 1932

Figures 11–20 , 26

Trioza alifumosa Klyver, 1932: 96.

##### Distribution.

Reported on the Cook Islands in the present study. Known only from Rarotonga. Other locations include: French Polynesia (Marquesas, Fatu Hiva) (Klyver 1932).

##### Host plant.

*Metrosideroscollina* (J.R. Forst. & G. Forst.) A. Gray (Myrtaceae).

#### Trioza vitiensis

Taxon classificationAnimaliaHemipteraTriozidae

Kirkaldy, 1907

Figure 29

Trioza vitiensis Kirkaldy, 1907: 103.
Megatrioza vitiensis (Kirkaldy); Crawford 1919: 195.
Phyllopecta vitiensis (Kirkaldy); Klyver 1932: 99.
Trioza vitiensis Kirkaldy, 1907 combinatio revivisco according to Mathur (1975): 348.

##### Distribution.

Reported on the Cook Islands by Hodkinson (1983). Known only from Rarotonga. Other locations include: Asia [China, India, Indonesia, Malaya, Malaysia, Philippines, Singapore, Sri Lanka (Hodkinson 1983, 1986)], Pacific Islands [Caroline Islands, Fiji, French Polynesia (Societies, Marquesas), Samoa (Kirkaldy 1907, Hodkinson 1983)].

##### Host plant.

*Syzygiummalaccense* (L.) Merr. & L.M.Perry, 1938 (Myrtaceae).

#### Trioza cf. zimmermani

Taxon classificationAnimaliaHemipteraTriozidae

Tuthill, 1942

Figure 24

##### Distribution.

Reported on the Cook Islands by P.J. Dale (McCormack 2007). Known only from Rarotonga.

##### Host plant.

*Metrosideroscollina* (J.R. Forst. & G. Forst.) A. Gray (Myrtaceae).

##### Remarks.

no specimens of this psyllid were collected by the authors. Photographs provided by G McCormack were consistent with the morphology of *T.zimmermani*, with the greatest difference shown in the wings (Figures 24, 27), with the Rarotongan specimens being shorter and with a less acute apex (Figure 24), than those from Raivaevae drawn by Tuthill (1942, Figure 27). However, since no specimens could be examined in person, this taxon is reported here based on the identification made by Dale. The distribution of *T.zimmermani* includes French Polynesia (Australs) (Tuthill 1942, Percy 2017).

**Figures 22–29.**
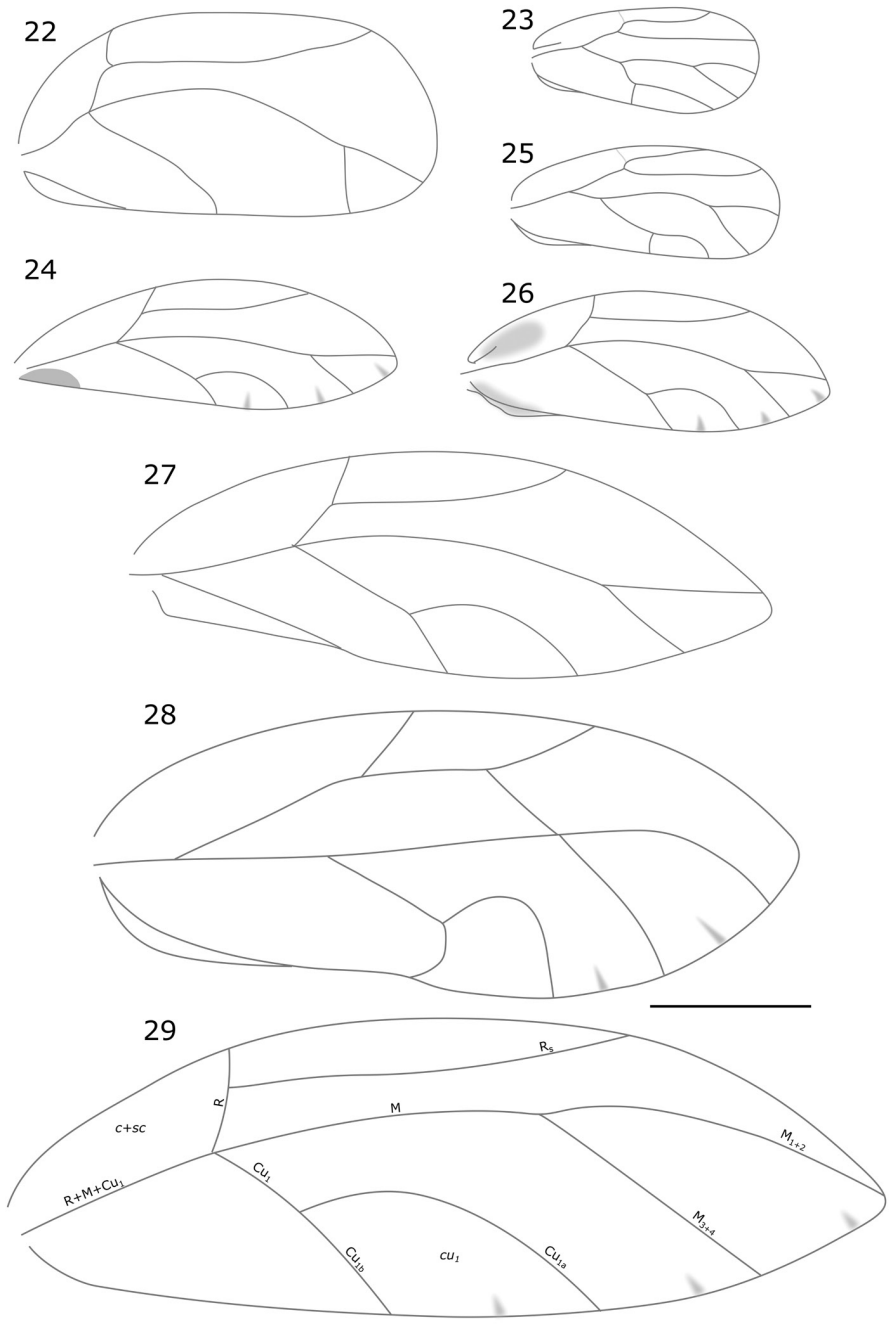
Wings, schematic. **22***Leptynopterasulfurea* (after Crawford 1919) **23***Syntomozatahuata* (from slide-mounted Rarotonga specimen) **24**Triozacf.zimmermani (from photograph of Rarotonga specimen by G. McCormack) **25***Heteropsyllacubana* (after Tuthill 1959) **26***Triozaalifumosa* (from slide-mounted Rarotonga specimen) **27***Triozazimmermani* (after Tuthill 1942) **28***Mesohomotomahibisci* (after Froggatt 1901) **29***Triozavitiensis* (after Klyver 1932). Scale bar: 1 mm.

### Key to the Cook Islands psyllids

**Table:** 

1	Forewing with vein R+M+Cu1 bi-furcating to form R and M+Cu1 (Figures 23, 25, 28)	**2**
–	Forewing with vein R+M+Cu1 tri-furcating to form R, M and Cu1 (Figures 24, 29)	**4**
2	Forewing with veins R and M+Cu1 equally long or M+Cu1 slightly longer than R (Figures 7, 23). Body colour black with orange stripes on the dorsum	***Syntomozatahuata* (Klyver, 1932)**
–	Forewing with vein R longer than M+Cu1 (Figures 25, 28). Body colour light green	**3**
3	Forewing with vein Rs very short (♂ 0.91, ♀ 1.14), strongly bent towards margin at apex, with a transverse vein crossing from centre of Rs to the bi-furcation between M1+2 and M3+4 (Figure 28)	***Mesohomotomahibisci* (Froggatt, 1901)**
–	Forewing with vein Rs not turning upward and no transverse vein crossing the wing (Figure 25)	***Heteropsyllacubana* Crawford, 1914**
4	Forewing with vein Cu1 not bi-furcating and therefore not forming cell cu1 (Figure 22). Body colour light brown	***Leptynopterasulfurea* Crawford, 1919**
–	Cell cu1 present (Figures 24, 29). Body colour darker brown/black	**5**
5	Forewing with dark spot on cell c+sc (Figures 17, 26). Body colour black with subtle brown patterning	***Triozaalifumosa* Klyver, 1932**
–	Forewing with no spots (Figures 24, 29). Body colour brown with tan pattern or black with pale stripe on the abdomen	**6**
6	Male genitalia with parameres pointing forward at apex and proctiger bearing long setae on the apical part facing the parameres. Female genitalia extremely short, approximately ¼ of abdomen. Length of psyllid to tip of folded wings between 5 mm and 6 mm. Body colour brown, with tan patterning	***Triozavitiensis* Kirkaldy, 1907**
–	Male parameres pointing backward at apex, proctiger bearing short setae uniformly, female terminalia longer (half of the rest of abdomen). Length of the psyllid to tip of folded wings only 3.5mm. Body colour black with pale stripe at base of abdomen	**Triozacf.zimmermani Tuthill, 1942**

## Discussion

Based on the similarity of the samples analysed with the description and the drawings provided by the literature, the presence of the psyllids *Syntomozatahuata* and *Triozaalifumosa* is reported on the Cook Islands for the first time. Host plants for these two species in the Cook Islands are hypothesised to be *Weinmanniasamoensis* or *Homaliumacuminatum* and *Metrosideroscollina* respectively, based on collection data. Percy’s collection of a large number of individuals of *S.tahuata* from *Weinmanniaparviflora* suggests this genus could be a true host plant (Percy, personal communication). However, we consider that *H.acuminatum* should remain under consideration as a possible host. No specimens of *W.samoensis* were seen near the Avana Valley site where *S.tahuata* was collected from *H.acuminatum*, and the elevation of the site is well below the lower elevational limit of *W.samoensis* (Sykes 2016). The number of *S.tahuata* observed during this collecting event was much greater than were captured, and were much more abundant than on the occasions when *S.tahuata* was beaten from *W.samoensis*. A search for immature stages of *S.tahuata* on both *H.acuminatum* and *W.samoensis* should be undertaken to differentiate between these host plant hypotheses or confirm whether *S.tahuata* is a generalist (Burckhardt et al. 2014).

We consider these two species to be indigenous to the Cook Islands, despite their not having been recorded here previously. The Cook Islands are underexplored entomologically, with relatively little collecting having been done in indigenous vegetation in particular. Moreover, these species were found in areas of relatively intact vegetation, with little human modification, which tend to be more resistant to invasive species (Brockerhoff et al. 2010). We hypothesise that further investigation of the psyllid fauna in other islands of Eastern Polynesia will locate these species there also, in areas where *Metrosideroscollina* and *Homalium* species may be found. However, this in itself would not provide sufficient evidence to distinguish between hypotheses of recent or distant arrival in the Cook Islands. In the absence of past collections, analysis of rapidly evolving DNA regions would be necessary to provide further data to infer the arrival of these species in the Cook Islands.

The psyllid fauna of the Cook Islands now includes seven psyllid taxa from five genera and four families. The addition of *S.tahuata* is not only the first report for the genus in the Cook Islands, but also for the family Liviidae.

Compared with the psyllid fauna of other nearby archipelagos, the Cook Islands appear to have a very similar psyllid biodiversity. In fact, the single taxon present in Niue (*H.cubana*) and three of the four taxa present in Tonga (*H.cubana*, *M.hibisci*, and *L.sulfurea*) are also present in the Cook Islands (Ouvrard 2018). Similarly, the psyllid fauna of French Polynesia lists eight species, four of which are in common with the Cook Islands: *M.hibisci*, *T.zimmermani*, *T.alifumosa*, and *S.tahuata* (Ouvrard 2018). On the other hand, the Cook Islands do not share any of the three taxa present in American Samoa (Ouvrard 2018). A recent review indicates that the biota of the Society islands in many cases show close sister-taxon relationships with the Cook, Austral, and Marquesas Islands (Hembry and Balukjian 2016). They also found that many taxa showed patterns of multiple colonisation of the islands, indicating high species turnover in the Eastern Polynesian region (Hembry and Balukjian 2016). We believe that the records of the two psyllid species reported here for the first time from the Cook Islands provides further evidence of the recognition of a distinctive Eastern Polynesian fauna.

## Supplementary Material

XML Treatment for Syntomoza tahuata

XML Treatment for Trioza alifumosa

XML Treatment for Mesohomotoma hibisci

XML Treatment for Syntomoza tahuata

XML Treatment for Heteropsylla cubana

XML Treatment for Leptynoptera sulfurea

XML Treatment for Trioza alifumosa

XML Treatment for Trioza vitiensis

XML Treatment for Trioza cf. zimmermani
